# Chronic Facial Paralysis Treated With Non-absorbable APTOS Barbed Threads: A Case Report With Anatomic Considerations

**DOI:** 10.7759/cureus.62539

**Published:** 2024-06-17

**Authors:** Merita Mazreku, Taras Feltsan, Hisham El Falougy, Katarina Bevizova

**Affiliations:** 1 Department of Stomatology and Maxillofacial Surgery, Faculty of Medicine and Oncologic Institute of St. Elisabeth, Comenius University, Bratislava, SVK; 2 Institute of Anatomy, Faculty of Medicine, Comenius University, Bratislava, SVK; 3 Institute of Anatomy, Faculty of Medicine, Comenius University in Bratislava, Bratislava, SVK

**Keywords:** esthetic restoration, anatomic considerations, non-surgical approach, aptos threads, facial nerve paralysis

## Abstract

Chronic paralysis of the facial nerve leads to degenerative facial muscle and surrounding soft tissue alterations on the involved side, making the affected patients seem older than their actual age. Moreover, contralateral facial hypertrophy worsens facial asymmetry. Correction of the drooping or wrinkled face due to aging, trauma, or other pathology has been successfully treated with the thread-lifting technique. Here, we present the case report of a 23-year-old female patient suffering from oncologic post-surgery complications associated with chronic facial nerve paralysis. She also suffered from old and new cerebellar syndromes on the right side and lesions of the oculomotor, trochlear, and abducens nerves. Based on the patient history, the condition was treated under local anesthesia by the use of APTOS minimally invasive threads with barbs made from non-absorbable material. Correction and sculpting of the affected cheek area were performed by insertion of a light lift needle, and lifting of the superficial fat pads was secured by subdermal insertion of the light lift thread method. The jowl area was lifted by the superficial insertion of both types of threads. As a result, we significantly improved facial symmetry at rest, a more symmetric smile, a lifted corner of the mouth, and an anatomically sculpted cheek appearance.

## Introduction

Chronic paralysis of *nervus facialis* leads to degenerative facial muscle alterations on the involved side, thus making the affected patients seem older than their actual age. Moreover, contralateral facial hypertrophy significantly worsens facial asymmetry. In many cases, it may result not only in aesthetic abnormality but also in lowered confidence levels, anxiety, depression, and social isolation [1,2].

The severity of facial paresis appearance depends on the original cause. It can be complete or partial, permanent or temporary [3]. Complete and temporary facial paresis usually occurs after trauma or iatrogenic causes after damaging the facial nerve during parotid gland, temporomandibular joints, and brain surgeries. Unilateral peripheral facial nerve paralysis may have an obvious cause (secondary facial nerve palsy) or may be idiopathic (primary) without a detectable cause (Bell’s palsy). Secondary facial nerve palsy is generally less prevalent than Bell’s palsy (25 vs. 75%) [4].

The facial asymmetry and sagging lead to the inability to smile, and the affected patient constantly has a displeasure facial expression. Moreover, functional impairment can affect eye movement, speech, and the ability to chew food. Chronic facial paralysis is associated with the degeneration of facial muscle fibers, which can cause eyebrow drooping, nasolabial fold flattening, the formation of jowls, and lip volume reduction.

As mentioned, morphological alterations are frequently accompanied by hemifacial synkinesis and contractures. Facial skin and soft tissues become loose and saggy, on the affected side, and the malar fat pad alights along the inferomedial direction. Overall, there is a significant volume loss on the affected side of the face [5].

Recently, the surgical treatment of facial paralysis has been divided into two groups: static and dynamic. The first approach is focused on restoring function, and the other one addresses the improvement of facial deformities. However, both approaches require major surgical interventions, both of which are extensively laborious and have insubstantial results [6,7]. A method of thread-lifting has been widely applied to improve a saggy or wrinkled face as a result of the aging process, trauma, or other pathology. As it is a simple and minimally invasive facial reconstruction technique, its popularity has been rising in recent years [8].

Herein, we present a challenging case report of a 23-year-old female patient suffering from oncologic post-surgery complications associated with chronic facial nerve paralysis. We also provide the most important anatomical aspects to reveal a complete understanding of the present technique.

Anatomic considerations

*Nervus facialis* (VII. cranial nerve) is the major motor nerve of the head, which consists of two parts: the motor root of the *nervus facialis* and the mixed *nervus intermedius*. The roots of the facial nerve leave the brainstem at the cerebellopontine angle. Upon exiting the brainstem, the *nervus facialis* and *nervus intermedius* join each other and transverse the *fossa cranii posterior*. On the posterior part of the petrous bone, it enters the porus and *meatus acusticus internus*. Then it passes through the *canalis nervi facialis* (also called the *canalis Fallopi*). The nerve exits the skull at the *foramen stylomastoideum*. The nerve stem descends from the *foramen stylomastoideum* for about 1 cm before curving anteriorly and penetrating the deeper part of the *glandula parotis* [9]. Branches of the nerve, so-called *plexus intraparotideus*, are positioned at a separate layer of the connective tissue between the superficial and deep portions of the *glandula parotis*. There are two main branches of the ​​​​​​​*nervus facialis* (the upper *ramus temporofacialis* and the lower *ramus cervicofacialis*) that are connected by this plexus. Terminal branches leave the parotid gland as *rami temporales* (forehead branches), *rami zygomatici*, *rami buccales*, *ramus marginalis mandibulae,* and *ramus coli nervi facialis* for the innervation of platysma and the mimic muscles of the face [10].

The fat pads of the cheek area are also important components of the face, and their layout, shape, and volume significantly influence the facial features. Anatomically, facial adipose tissue can be divided into superficial and deep compartments [11]. Superficial fat is located between the inner skin surface and the superficial musculoaponeurotic system (SMAS). It’s formed by fat pads, which are separated by septa of connective tissue. These septa extend between the skin and the SMAS and divide the cheek fat into smaller compartments: the nasolabial compartment, the medial cheek fat compartment, the middle cheek fat compartment, the lateral temporal cheek fat compartment, and the jowl fat compartment. The deep fat compartment of the cheek is formed by a deep cheek fat pad. The deepest adipose tissue creates the buccal (Bichat) fat pad, which is located between the *musculus masetter* and the *musculus buccinator* [12].

SMAS is often defined as an organized fibrous network created of the platysma, fascia parotis, and fibromuscular layer, which cover the cheek and neck. It connects the facial muscles with the dermis. This network also separates the subcutaneous fat from the *fascia parotideomasseterica* and the branches of the *nervus facialis* [13,14]. Anatomically, the boundaries of the SMAS are the *arcus zygomaticus* superiorly and the *platysma* inferiorly. The subcutaneous superficial adipose tissue of the face is located anteriorly to the SMAS, and the parotidomasseteric fascia is located beneath. 

The retaining ligaments of the cheek area are strong and deep fibrous connections that originate from the periosteum or deep facial fascia and extend vertically through facial layers to insert into the dermis [15]. Anatomically, retaining ligaments are divided into true and false. The true retaining ligaments are strands of connective tissues that insert into the bone. The false retaining ligaments extend between muscles and connective tissue of the skin and connect muscles and muscles with the skin [10]. Their relationship to facial nerve branches is clinically particularly important.

The zygomatic ligament (McGregor´s patch) is defined as an adherent area over the malar eminence that attaches the skin of the cheek to the inferior border of the zygomatic bone just posterior to the origin of *musculus zygomaticus minor* [16].

The temporoparotid fascia, so-called Lore´s fascia, is usually defined as a band running from the *fissura tympanomastoidea* to the *glandula parotis*, with the main trunk of the facial nerve located directly beneath it [17].

## Case presentation

**Figure 1:**
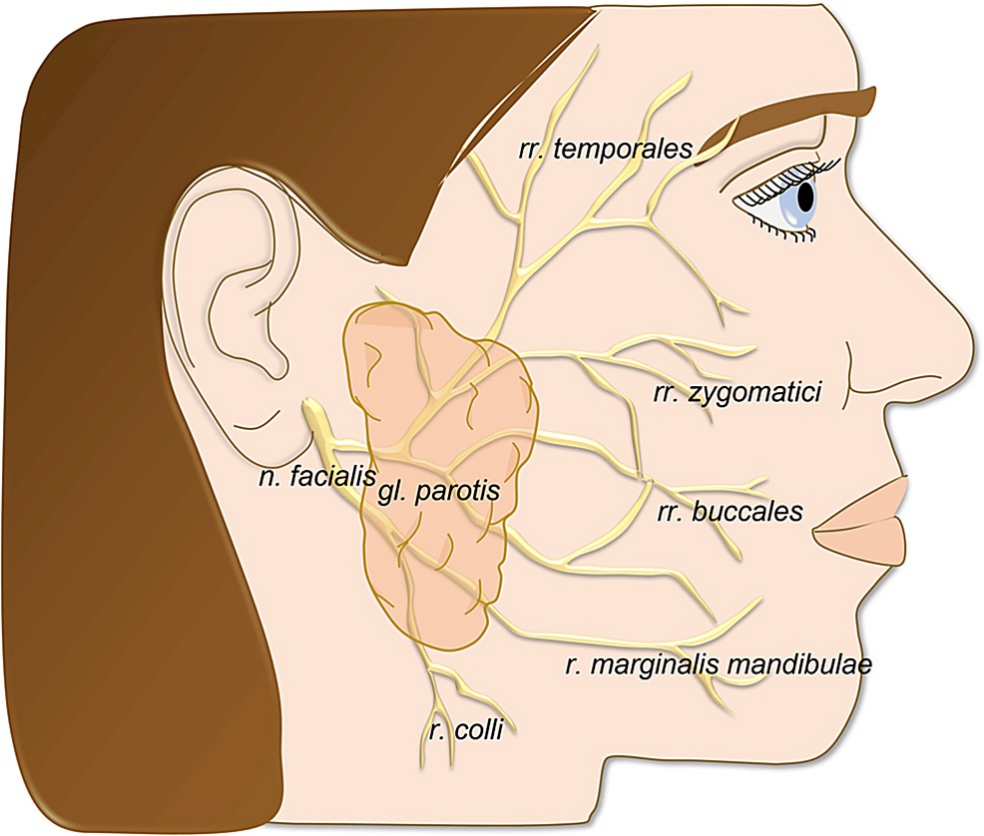
Nervus facialis. The presented figure is original artwork.

Figure 1 shows the nervus facialis.

**Figure 2:**
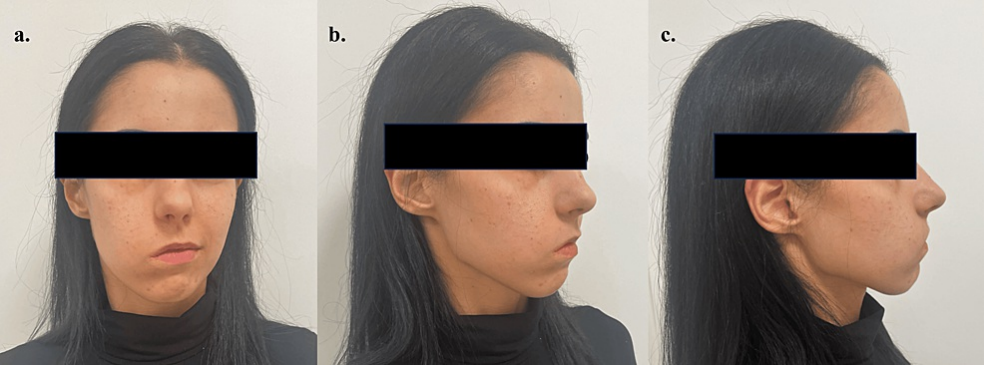
Preoperative appearance of the patient.

A 23-year-old female patient presented to our clinic with a previous diagnosis of astrocytoma of the fourth brain chamber and underwent tumor resection surgery in 2008. The patient developed postoperative complications associated with chronic facial nerve paralysis. She also suffered from paleo- and neocerebellar syndrome on the right side and lesions of the *nervus oculomotorius*, *nervus trochlearis*, and *nervus abducens*. A physical examination revealed a significant facial asymmetry (Figure 2), both at rest and during facial expressions; the nasolabial fold was smoothed out, and the right corner of the mouth lags behind facial expressions. A slight asymmetry of the eye slits, lag ophtalmus, oculomotor disorder, and nystagmoid involuntary eye movements when looking to the left were also detected. Furthermore, in the extinction positions, slight instability of the right limbs and dysmetria to the right were observed. Stance and walking on a wider base with lateral titubation more pronounced to the right. Written informed consent was obtained from the patient. The principles of the 1975 Declaration of Helsinki were followed.

**Figure 3:**
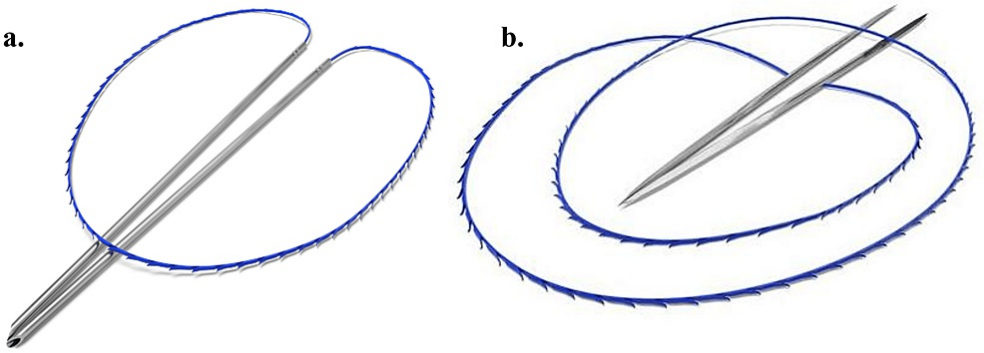
APTOS light lift thread 2G (LLT) and APTOS light lift needle (LLN) 2G.

**Figure 4:**
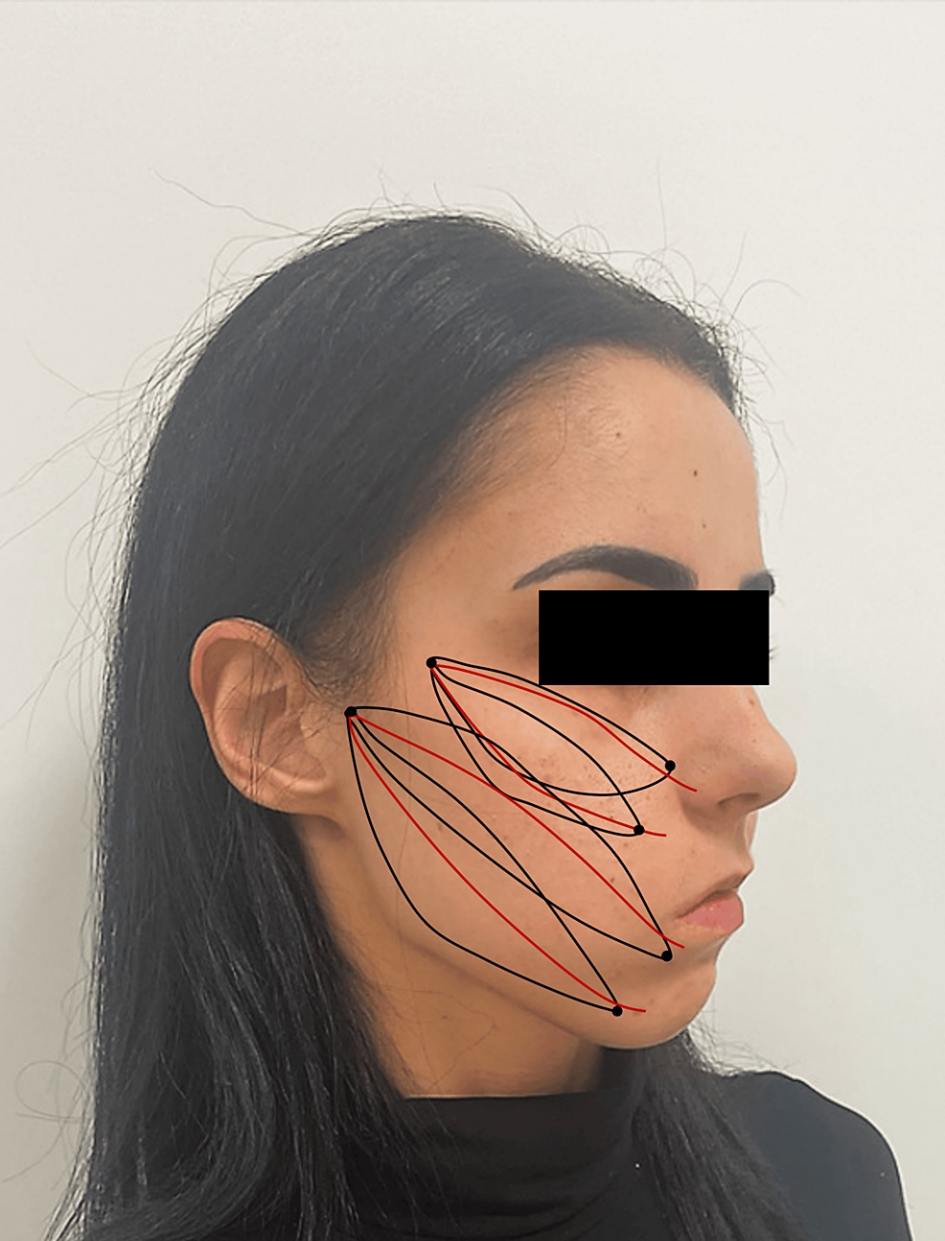
Application of threads.

Based on the patient´s history and results of the physical examination, we considered using APTOS minimally invasive threads with barbs made from non-absorbable material: APTOS Light Lift Thread 2G (LLT) and APTOS Light Lift Needle (LLN) 2G (Figure 3). The procedure was performed after the infiltration administration of the local anesthetic Supracain 4% (Zentiva, Slovakia) by a blunt tip cannula (22G, 7 cm) into subcutaneous tissues. Correction and sculpting of the affected cheek area were performed by insertion of the LLN 2G thread, with the insertion point placed in the area of McGregor’s patch, which was also used as an exit point. A thread was inserted in the deep fat pads of the perimaxillar area to create cheek volume and sculpt the nasolabial region while turning the needle and creating a loop in the area of the flattened nasolabial fold. We lifted the superficial fat tissue, inserting LLT 2G at the same entry point, not undergoing SMAS, and exiting in the sculpted nasolabial fold. Fixation of the thread was also secured in the area of McGregor’s patch. The procedure was followed by lifting the lip corner and jowling soft tissues by subdermal insertion of LLN and LLT with the insertion in the preauricular region and fixation on Lore’s fascia. The loop under the corner of the mouth, created above the *musculus depressor anguli oris,* helps to treat mouth asymmetry by traction of tissues and also by creating a volume above the muscle (Figure 4).

After the procedure, the patient received a bandage and was instructed about the necessity of adhering to the aftercare actions (10 days of mushy diet, 10 days of no sports activity, and regular application of Fast recovery cream (Recowell®, Russia).

**Figure 5:**
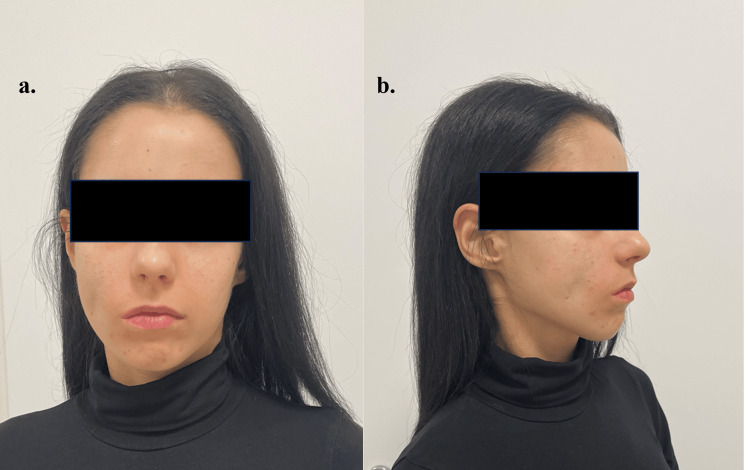
Result after 10 days: a significant improvement in facial symmetry was achieved.

As a result, after 10 days, a significant improvement in facial symmetry at rest, a more symmetric smile, a lifted corner of the mouth, and an anatomically sculpted cheek appearance were noticed (Figure 5). The patient did not report pain or other discomfort. A full recovery was achieved after 14 days.

## Discussion

Trauma, including iatrogenic damage to the facial nerve, represents 10-23% of cases, and it is the second most common reason for facial paralysis [18]. It leads to numerous adverse effects, such as facial asymmetry due to the loss of mimic muscle tone on the affected side, reduced control of facial movements, and difficulties in emotional expression that significantly decrease quality of life [2]. The main goals of the treatment of facial paralysis include facial symmetry at rest, a symmetrical smile, voluntary, coordinated, spontaneous facial movements, oral competence, eyelid closure with corneal protection, and the absence or limitation of synkinesis and mass movement [19].

Various surgical and nonsurgical approaches have been adopted for facial correction, rejuvenation, and beautification. Surgical techniques have been widely used in reconstructive and aesthetic surgery to achieve restoration of facial movement and support soft tissues in the face. Surgery represents a burden to patients; in many cases, it is time-consuming and may be associated with an increased risk for adverse effects such as pain, bleeding, swelling, hematomas, seromas, prolonged wound healing, sepsis, etc. [7,20]. Also, nonsurgical procedures are used, including botulotoxin A and various filler applications [21,22]. However, the mentioned procedures often do not bring the expected result.

Another minimally invasive, simple, and time-saving approach using non-absorbable threads (APTOS methods) was introduced by Sulamanidze et al., who performed facial lifting [23]. They obtained very promising results in lifting various ptotic areas of the face. The improvement persisted in most patients with a follow-up of two to 30 months. Later, it was proven that this type of thread is ideal for use in patients with uncurable types of facial paralysis. The indications for applying the APTOS methods include facial asymmetry, more than one year of facial paresis without improvement, electromyoneurography data showing the absence of electrical and graded potential, and the need for correction after surgeries [24]. In our case, we used absorbable types of threads because their application stimulates collagen production by fibroblasts in the dermis and subdermal tissues, resulting in a stronger lifting effect that supports facial muscles by the time of their full recovery [25].

Potential limitations of correction by using lifting threads consist of only static improvement of the face; therefore, strong facial expressions remain asymmetric. On the other hand, better dynamic results could be achieved by combining static tissue lifting using threads with the myomodulative effect of botulinum toxin applied to mimic muscles on the contralateral side [26]. Another complication may be associated with the tightening of the skin a few months after inserting lifting threads in the subdermal tissue and may appear due to the fibrosis process followed by permanent contraction of fibrous tissue and subsequent traction on the skin [27].

## Conclusions

In conclusion, it can be emphasized that APTOS non-absorbable threads' distinctive design improves facial symmetry and functionality. Subcutaneous tissue repositioned with APTOS threads can be given elevation, fixation, and the ability to create volume in the tissues of affected areas. 
